# Weight loss and brown adipose tissue reduction in rat model of sleep apnea

**DOI:** 10.1186/1476-511X-7-26

**Published:** 2008-07-31

**Authors:** Denis Martinez, Luiz FT Vasconcellos, Patricia G de Oliveira, Signorá P Konrad

**Affiliations:** 1 Division of Cardiology, Hospital de Clinicas de Porto Alegre, Universidade Federal do Rio Grande do Sul, Brazil

## Abstract

**Background -:**

Obesity is related to obstructive sleep apnea-hypopnea syndrome (OSAHS), but its roles in OSAHS as cause or consequence are not fully clarified. Isocapnic intermittent hypoxia (IIH) is a model of OSAHS. We verified the effect of IIH on body weight and brown adipose tissue (BAT) of Wistar rats.

**Methods:**

Nine-month-old male breeders Wistar rats of two groups were studied: 8 rats submitted to IIH and 5 control rats submitted to sham IIH. The rats were weighed at the baseline and at the end of three weeks, after being placed in the IIH apparatus seven days per week, eight hours a day, in the lights on period, simulating an apnea index of 30/hour. After experimental period, the animals were weighed and measured as well as the BAT, abdominal, perirenal, and epididymal fat, the heart, and the gastrocnemius muscle.

**Results:**

Body weight of the hypoxia group decreased 17 ± 7 grams, significantly different from the variation observed in the control group (p = 0,001). The BAT was 15% lighter in the hypoxia group and reached marginally the alpha error probability (p = 0.054).

**Conclusion:**

Our preliminary results justify a larger study for a longer time in order to confirm the effect of isocapnic intermittent hypoxia on body weight and BAT.

## Introduction

Sleep apnea is a threatening aftermath of the obesity epidemics [1]. Obstructive apneas start with the occlusion of the pharynx after sleep onset, progress during 10 to 40 seconds with increasing asphyxia, and end with an arousal [2]. The episodes can be repeated hundreds of times leading to consequences via sleep fragmentation and intermittent hypoxia (IH). IH can trigger inflammation and the release of free radicals [3,4]. Although the data are conflicting [5,6], hypoxia seems to be the main factor causing leptin increase in obstructive sleep apnea-hypopnea syndrome (OSAHS) patients [7]; since leptin is an appetite-suppressing and metabolism-enhancing protein hormone, the expected effect of such finding would be weight loss in OSAHS. Weight reduction is associated with improvements in sleep apnea pointing towards an etiologic role of obesity [8,9]. Obesity may be dually associated to obstructive apneas, acting as a cause, by narrowing the pharynx [10,11] and as a consequence of apnea-related sleep deprivation [12,13].

The brown adipose tissue (BAT) [14] is an organ with the specialized function of nonshivering thermogenesis, through intracellular fat oxidation. Recently, it was described that a significant percentage of adult human population display activity compatible with the existence of brown adipose tissue potentially significant for metabolic physiology [15]. The uncoupling protein 1 (UCP1) existent in BAT cells makes possible for the mitochondria to release energy directly in heat form [16]. The metabolic activity necessary for the muscles to produce the same heat would require larger blood flow and oxygen consumption rate. The UCP1 expression is regulated by catecholamines, thyroid hormones and leptin and occurs, for example, during the exposure to cold [17], in the postnatal period, in febrile stages or during hibernation [18].

Hypoxia induces body temperature reduction in mammals mediated by the central nervous system, bringing about decrease of oxygen consumption and increase of hemoglobin-oxygen affinity. This adaptation may affect the BAT activity. In rats, this body temperature decrease could be mediated by the inhibition of cutaneous vasoconstriction or of nonshivering thermogenesis [19]. The metabolism and heat production in BAT are primarily controlled by sympathetic impulses [20] and, during nonshivering thermogenesis, more than a half of all the oxygen captured is consumed by BAT [18].

Hypoxia stimulates the carotid body arterial chemoreceptors, whose fibers found in the carotid sinus activate neurons of the solitary tract nucleus, enabling the hypothalamus to control temperature through the autonomic nervous system. Activation of the afferent arterial chemoreceptors during brief hypoxia episodes increases the sympathetic outflow [21].

Models of intermittent hypoxia are used in animals and in cell cultures to demonstrate anatomic and functional alterations, in sympathetic reactivity to hypoxia and hipercapnia [22,23]. Studies that submit animals to short cycles, alternating hypoxia and normoxia, preferably maintaining isocapnic condition, at every 30 to 90 seconds, during sleep, between 7 and 8 daily hours are experimental models of sleep apnea. To the best of our knowledge, no study using a model that simulates OSAHS has investigated the effect of isocapnic intermittent hypoxia (IIH) on the adipose tissue. Our study verified the effect of IIH, simulating sleep apnea, on the adipose tissue of adult Wistar rats.

## Methods

Nine-month-old male breeders Wistar rats, recently removed from breeding, were separated in two groups: 8 rats submitted to 21 days of IIH and 5 control rats, submitted to 21 days of sham IIH, both groups housed under 22°–24°C and *ad libitum *fed. The protocol was approved by the local Ethics Committee. The rats were weighed at the baseline and at the end of 21 days, after anesthesia, prior to sacrifice. The nose-anus length was measured with a ruler and rounded to the next centimeter. The Lee index was calculated as the division of the body weight cubic root in grams, by the nose-anus length in millimeters, multiplied by 10 [24].

During three weeks, 7 days per week, for 8 hours a day, from 9 a.m. to 5 p.m., in the lights on period, the rat cages were covered with transparent acrylic lids connected to the IIH system. Food and water supplies were kept unchanged during the experimental procedure. A timer activated a solenoid valve to release a mixture with 92% of nitrogen and 8% of CO_2 _in the hypoxia chamber, for 60 seconds. At the end of this time interval, the nitrogen flow in the cages had reduced the oxygen fraction from 21% to approximately 7 ± 1% and increased the CO_2 _fraction to approximately 5 ± 1%, lasting the nadir 12 ± 3 seconds. At the end of the hypoxia cycle, the timer activated a fan for 60 seconds insufflating room air into the chamber, restoring the oxygen fraction to 21%, in 30 ± 5 seconds, and conducting the chamber air out of the room. In 120 seconds, there was a full hypoxia/normoxia cycle; in 8 hours, 240 IIH periods occurred, the equivalent to an apnea index of 30 per hour, a figure commonly observed in patients with severe OSAHS.

In order to confirm the arterial blood oxygen saturation (SaO_2_) level reached in the animals submitted to this equipment, two anesthetized rats had the SaO_2 _measured through a pulse oximeter for veterinary use (NONIN Medical, Inc., Plymouth, Minnesota, USA). Repeated arterial saturation falls were verified to values around 70%, SaO_2 _level usually observed in patients with severe OSAHS.

**Figure 1:**
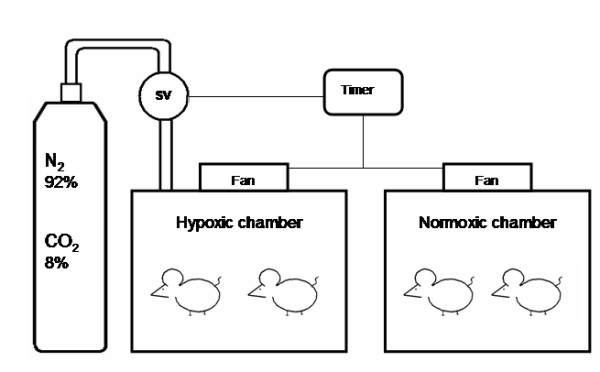
**Diagram of the hypoxic and normoxic chambers**. Solenoid valve (SV).

The sham IIH group remained in an adjacent cage and underwent the same handling as the IIH group, but the normoxia chamber was insufflated with air instead of N_2 _+ CO_2_. The purpose was to control for the stress arising from cover installation on cages and from fan and gas release noises. After three weeks, eight animals from the IIH group and five from the sham IIH were anesthetized for organs removal. Wet weight of white AT (abdominal and epididymal), heart, and gastrocnemius muscle was measured. The BAT was removed and weighed immediately after that in the two groups. Body composition was determined by evaluating the fat mass, represented by the weight in grams of the abdominal adipose white tissue (epididymal, perirenal and mesenteric) and the interescapular BAT and the lean mass, represented by the sum of the weight in grams of the gastrocnemius skeletal muscle and the cardiac muscle, all of them removed and weighed immediately after sacrificing the animals (Figure 1).

### Statistical Analysis

Results are expressed as mean ± SD. To compare paired observations of weights before and after 21 days we used Willcoxon exact test. To compare IIH versus sham IIH we employed Mann-Whitney's U with Fisher's exact one-tailed test.

## Results

As shown in Table 1, the average body weight of the animals from the IIH group decreased after 21 days, while it did not change in the sham IIH group. We did not find difference in heart dimensions. The weight loss and the reduction in the Lee index in the IIH group were significantly different from the variation observed in the sham IIH group (p = 0,001). The BAT was lighter in the IIH group and marginally reached the alpha error probability (p = 0.05) (Table 1).

**Table 1:** Mean and standard deviations of body weight, Lee Index and body fat distribution in the two groups of animals prior and after the isocapnic intermittent hypoxia (IIH) or sham IIH exposure

	**IIH** (n = 8)	**Sham IIH** (n = 5)	**P**
**Initial body weight**	446.75 ± 50.31	448.40 ± 62.67	0.362*
**Final body weight**	429.50 ± 52.88	451.60 ± 60.71	0.262*
**Initial – final weight**	17.25 ± 6.69	-3.20 ± 8.17	0.001**
**Nasal – anal length**	23.8 ± 0.8	23.4 ± 0.9	0.267*
**Initial Lee index**	0.320 ± 0.014	0.326 ± 0.015	0.111*
**Final Lee index**	0.315 ± 0.013	0.327 ± 0.014	0.015*
**Initial-final Lee index**	0.00428 ± 0.00187	-0.00079 ± 0.00188	0.001**
**Gastrocnemius**	1.04 ± 0.27	1.07 ± 0.17	0.308*
**Heart**	1.23 ± 0.137	1.24 ± 0.132	0.472*
**Brown adipose tissue**	0.51 ± 0.079	0.6 ± 0.075	0.054*
**Epididymal fat**	8.44 ± 2.558	9.65 ± 3.807	0.311*
**Abdominal fat**	19.58 ± 8.247	24.08 ± 16.048	0.528*

**Mann-Whitney; exact one-tailed test; * Willcoxon test.

## Discussion

The results of the present study suggest that 30 IIH cycles per hour, 8 hours a day, during 21 days cause a reduction in body weight and brown adipose tissue of Wistar middle-age retired breeder rats. The finding of body mass loss and BAT reduction in rats submitted to IIH is original. Despite the reduced number of cases, the BAT difference almost reaches statistic significance. Prior to reaching any definitive conclusions, this study should be repeated in a larger number of animals exposed to intermittent hypoxia for longer periods.

Before engaging in the present study, we performed a pilot study to collect evidence of an effect of IH on BAT. We measured the wet weight of BAT of mice being euthanasied after five weeks in IIH for another research protocol (unpublished data) to serve as a pilot. We found in 8 controls an average BAT wet weight of 0.0655 ± 0.005 g and in the 16 mice of the IIH group an average of 0.0334 ± 0.001 g, approximately half of the control group value (p < 0.0001). Because mice have a low total weight and minuscule BAT, we were concerned about errors occurring during dissection of BAT and decided to do this protocol with rats that are substantially larger. Speculating on what could account for a more prominent effect of IH in the mouse model (50% reduction) than in the rat (15% reduction), two possibilities are of interest: first, mice were submitted to 35 days of IIH against 21 days of the rats; second, the easier dissection of the BAT in rats made the results more exact. The latter is less likely since the variation coefficient is less important in the mice. Our initial experiences suggest that future research concentrate on species differences and on the effects of IIH duration on BAT.

The noradrenergic stimulus caused by IIH is the possible determinant of BAT consumption, considering that IIH raises the serum levels of sympathetic neurotransmitters [25]. Interescapular BAT in the rat is densely innervated by the sympathetic nervous system for the control of the temperature in cold [26] and diet-induced thermogenesis. Nonshivering thermogenesis performed by BAT is stimulated by hypoxia [27]. In acute hypoxic conditions, the O_2 _consumption response in cold is reduced or thoroughly suppressed by the shivering or nonshivering thermogenesis hypoxic depression. The BAT activity hypoxic depression mechanisms are not elucidated, although it is known that, even in young animals, the thermogenesis inhibition does not result from oxygen limitation, but from the response regulated by hypoxia [28].

Wet weight is a more relevant physiological parameter for white adipose tissue than for BAT. BAT wet weight may decrease both in face of increased and decreased tissue activity. To elucidate what causes the decrease in BAT it will be necessary to measure the amount of UCP1 or at least the protein amount in the BAT in future experiments.

Sleep was not recorded in this experiment; therefore, it is difficult to ascertain that the animals were sleep deprived. The overall effect of sleep fragmentation on total sleep time of OSAHS patients is still obscure. In a IH study by Veasey et al., in which sleep was recorded, mice increased sleep time significantly, indicating sleepiness; while the group submitted to two weeks of sham IH slept around 680 minutes per day, the IH group slept 900 minutes [29]. For the sake of reasoning, assuming that sleep time increased, the probability that leptin was increased is minimal. It is necessary, therefore, that leptin levels are measured to control for this confounding factor and to elucidate the effect of sleep fragmentation on leptin production.

We chose 9-month old retired breeder male rats as subjects in our protocol because these animals better simulate the middle-aged, sexually active men who suffer from OSAHS. There is some evidence that breeder male rats show responses to oxidative stress that are different from males that did not engage in reproductive activity [30].

Obesity is closely related to OSAHS, but until the present moment, obesity roles in OSAHS are not fully clarified: as predisponent factor, as consequence, or as both. If this study's findings are confirmed, the significant weight loss observed in the IIH group may be mediated by leptin increase and is in keeping with findings of correlation between hypoxia and leptin levels [7]. One interesting model to further the understanding of the effect of IIH on leptin is under way in our laboratory, repeating this experiment in leptin knockout obese mice.

This is the first report of weight loss and reduction of BAT under intermittent hypoxia. This line of research is warranted since the findings raised numerous questions that may be of relevance for sleep apnea patients. We recognized the need of additional studies in different species with larger number of animals and with longer expositions in order to confirm the effect of isocapnic intermittent hypoxia on body weight and BAT.

## Abreviations

BAT: Brown adipose tissue; IH: Intermittent hypoxia; IIH: Isocapnic intermittent hypoxia; OSAHS: Obstructive sleep apnea-hypopnea syndrome; SV: Solenoid valve; UCP1: Uncoupling protein 1.

## Competing interests

The authors declare not having any personal or financial support or involvement with organizations with financial interest in the subject matter or any actual or potential conflict of interest.
